# Validation of barley *2OGO* gene as a functional orthologue of Arabidopsis *DMR6* gene in *Fusarium* head blight susceptibility

**DOI:** 10.1038/s41598-020-67006-5

**Published:** 2020-06-18

**Authors:** Yee Chen Low, Michael A. Lawton, Rong Di

**Affiliations:** Department of Plant Biology, Rutgers University, New Brunswick, NJ USA

**Keywords:** Biotechnology, Plant sciences

## Abstract

*Fusarium* head blight (FHB) caused by *Fusarium graminearum* (*Fg*) is a devastating disease of crops, especially wheat and barley, resulting in significant yield loss and reduced grain quality. *Fg* infection leads to the production of mycotoxins, whose consumption is toxic to humans and livestock. The Arabidopsis *DMR6* gene encodes a putative 2-oxoglutarate Fe(II)-dependent oxygenase (*2OGO*) and has been identified as a susceptibility factor to downy mildew. We generated site-specific mutations in Arabidopsis *At2OGO* by CRISPR/Cas9 gene editing. The resulting *At2OGO* knock-out (KO) mutants display enhanced resistance to *Fg* in a detached inflorescence infection assay. Expression profiling of defense genes revealed that impairment of *At2OGO* function resulted in the upregulation of defense genes that are regulated by salicylic acid (SA), jasmonic acid (JA) and ethylene (ET) pathways. Complementation of the *At2OGO*-KO lines with a barley (cv. Conlon) orthologue, *Hv2OGO*, restored susceptibility to *Fg*. This result indicates that the *Hv2OGO* gene is functionally equivalent to its Arabidopsis counterpart and, hence, may have a similar role in conditioning susceptibility to FHB in barley. These results provide a molecular basis for proposing *2OGO* as a plant immunity suppressor in Arabidopsis and potentially in barley plants and establish a rationale and strategy for enhancing FHB resistance in barley.

## Introduction

*Fusarium* head blight (FHB) or scab is primarily caused by *Fusarium graminearum* (*Fg*) (teleomorph *Gibberella zeae*) and is a devastating disease of cereal crops, particularly wheat, barley and maize. FHB disease has been identified as a major limiting factor in cereal crop production throughout the world^1^. *Fg* is an important hemibiotrophic fungal pathogen whose growth is favored by warm conditions and high humidity and whose dispersal is aided by frequent rainfall during flowering season^2^.The most noticeable disease symptom of FHB is bleaching of floral spikelets, which often leads to cell death or the production of non-viable seeds^3^. Since the early 1990s, it has been estimated that FHB infection in grain crops exerts an annual loss of US$3 billion^4,5^. In addition to yield reduction, FHB also lowers grain quality by producing mycotoxins, including deoxynivalenol (DON), which accumulate in infected grains^6,7^. DON-contaminated grains represent a serious health concern for both humans and livestock. Deployment of FHB-resistant wheat and barley cultivars provides the most direct way to address this problem as it simultaneously reduces yield loss and grain contamination.

Genetic engineering offers a promising approach for manipulating plant disease resistance and susceptibility. A number of different transgenic approaches have been explored in order to generate plants that display enhanced FHB resistance or tolerance. These include the overexpression of pathogenesis-related (PR) or defense response genes^8–10^, overexpression of antifungal^11^ or antimicrobial peptides^12^ introduced from other species, the inhibition of DON synthesis^13–15^, modifying the host cellular target of DON^16^ and detoxifying DON to less toxic compounds^17,18^. These transgenic plants are considered to be genetically modified organisms (GMO), which may confer an undue regulatory burden on producers and processors as well as creating a barrier to consumer acceptance. The emergence of CRISPR technology allows precise and efficient genome editing by introducing mutations at specific target sites^19^. CRISPR has been used intensively in mammalian cells and many whole organisms^20–24^ including both model and crop plants^25–32^. The potential for generating transgene-free and gene-edited disease resistant plants by CRISPR gene editing prompted us to use CRISPR technology to generate plants with enhanced FHB resistance^33–35^.

Identification of host susceptibility genes provides a foundation for developing disease resistant plants through genome editing. A susceptibility gene called *DMR6* (downy mildew resistance 6) was discovered and characterized while screening Arabidopsis ethyl methane sulfonate and T-DNA insertion mutants for the loss-of-susceptibility to downy mildew disease caused by the *Hyaloperonospora arabidopsidis*^36,37^. Map-based gene cloning revealed that *DMR6* encodes a putative 2-oxoglutarate Fe(II)-dependent oxygenase (2OGO) that belongs to the 2-oxoglutarate-dependent dioxygenase superfamily. The *dmr6-1* null and *dmr6-2* T-DNA mutations result in incorrect intron splicing and the resulting inability to produce a functional 2OGO protein. Such mutants display a decreased susceptibility to downy mildew and this is thought to result from the observation that *2OGO* is a negative regulator of defense-associated genes. It was later shown that *DMR6* and its paralog *DLO1* (*DMR6-like oxygenase 1*) were co-expressed during pathogen infection and redundantly suppressed plant immunity through a mechanism of salicylic acid (SA) homeostasis^36^. Hence, mutations in *DMR6* and *DLO1* resulted in an increase of SA levels and the upregulation of *pathogenesis related (PR)-1*, *PR-2* and *PR-5* genes. Interestingly, the *dlo1* mutant showed a lower level of resistance to *H. arabidopsidis* compared to the *dmr6* mutant, whereas, the *dmr6dlo1* double mutant showed complete resistance to downy mildew, albeit with an accompanying dwarf phenotype. It was concluded that *dmr6* mutant alone provides broad spectrum disease resistance to *H. parasitica*, *Pseudomonas syringae* and *Phytophthora capsici*^38^. It was also shown the *dmr6* mutant was resistant to *Fusarium culmorum*^39^. The biochemical mechanism of *DMR6* was further elucidated by a recent report^40^ showing that *DMR6* encodes a salicylic acid 5-hydroxylase (S5H) which catalyzes the conversion of SA to 2,5-dihydroxybenzoate (2,5-DHBA) by hydroxylating SA at the C5 position of its phenyl ring. It was shown that the *DMR6/S5H* expression is induced by SA and that conversion of SA to 2,5-DHBA provides a feedback mechanism which maintains SA homeostasis in Arabidopsis cells. Considering the broad-spectrum resistance to different pathogens induced by *DMR6/S5H/2OGO* mutation, generating CRISPR-edited *dmr6* mutants represents a promising solution to restrict diseases in crop plants.

Arabidopsis has been shown to be infected by *F. graminearum* and used to study the interaction between the host plant and the pathogen^41,42^. In this study, we generated *2OGO*-knock out (KO) mutant Arabidopsis plants using the CRISPR/Cas9 system. The CRISPR-edited Arabidopsis *2OGO*-KO mutants carry frameshift mutations that are predicted to produce non-functional 2OGO enzymes. These edited plants, termed *At2OGO*-KO, show enhanced resistance to *Fg* in inflorescence tissues while remaining normal with respect to plant growth and development. We also identified a *2OGO* orthologue of barley (cv. Conlon) and introduced the corresponding *Hv2OGO* cDNA into an Arabidopsis *2OGO*-KO mutant line. This complementation experiment fully restored susceptibility of Arabidopsis to *Fg*, indicating that *Hv2OGO* is able to function as a suppressor of FHB resistance in Arabidopsis, most likely through conserved biochemical and molecular mechanisms. This result suggests that *Hv2OGO* may play a similar role in conditioning FHB susceptibility and other infectious diseases in barley. These studies provide a foundation for the creation of CRISPR/Cas9-edited barley plants that are similarly and significantly enhanced for resistance to FHB.

## Results

### Generating *2OGO*-KO (*dmr6*) mutants in Arabidopsis using CRISPR/Cas9 system

*2OGO* genomic (*At5g24530*) and mRNA (NM_122361.4) sequences were obtained from the TAIR and NCBI databases respectively. The active site of *2OGO* (oxoglutarate/iron-dependent dioxygenase domain), which resides within the C-terminal portion of its protein sequence (from a.a.188 to a.a.288) corresponds to exon 3 of the genomic sequence and is responsible for the *2OGO*-dependent susceptibility phenotype in *A. thaliana*. We designed a guide RNA (gRNA) that specifically targets exon 3 (target region: 5′- GGTCTCCAGATCTTGATCGA-3′) and that also flanks a *Bgl*II restriction site (Fig. 2, underlined) which could be used for mutant screening. The gRNA target was ligated into the psgR-At vector (kindly provided by Dr. J. Zhu^28^) which contains an Arabidopsis U6 promoter to express the gRNA and a Cas9 gene whose expression is controlled by the 2 × 35 S promoter and the nopaline synthase (NOS) terminator. For *Agrobacterium tumefaciens* transformation, the gRNA/Cas9 cassettes were subcloned into *Eco*RI- and *Hind*III-digested pCAMBIA1300, resulting in the plant expression vector pRD207. Flowering Arabidopsis Col-0 plants were transformed *via Agrobacterium*-mediated transformation using *Agrobacterium* strain EHA105 and the floral-dip Arabidopsis transformation method. The collected T_0_ seeds were screened for germination on 50 μg/L hygromycin. A total of 14 putative T_1_ mutant plants were recovered from antibiotic selection, all of which were phenotypically similar to the WT plant and produced viable seeds.

**Figure 1:**
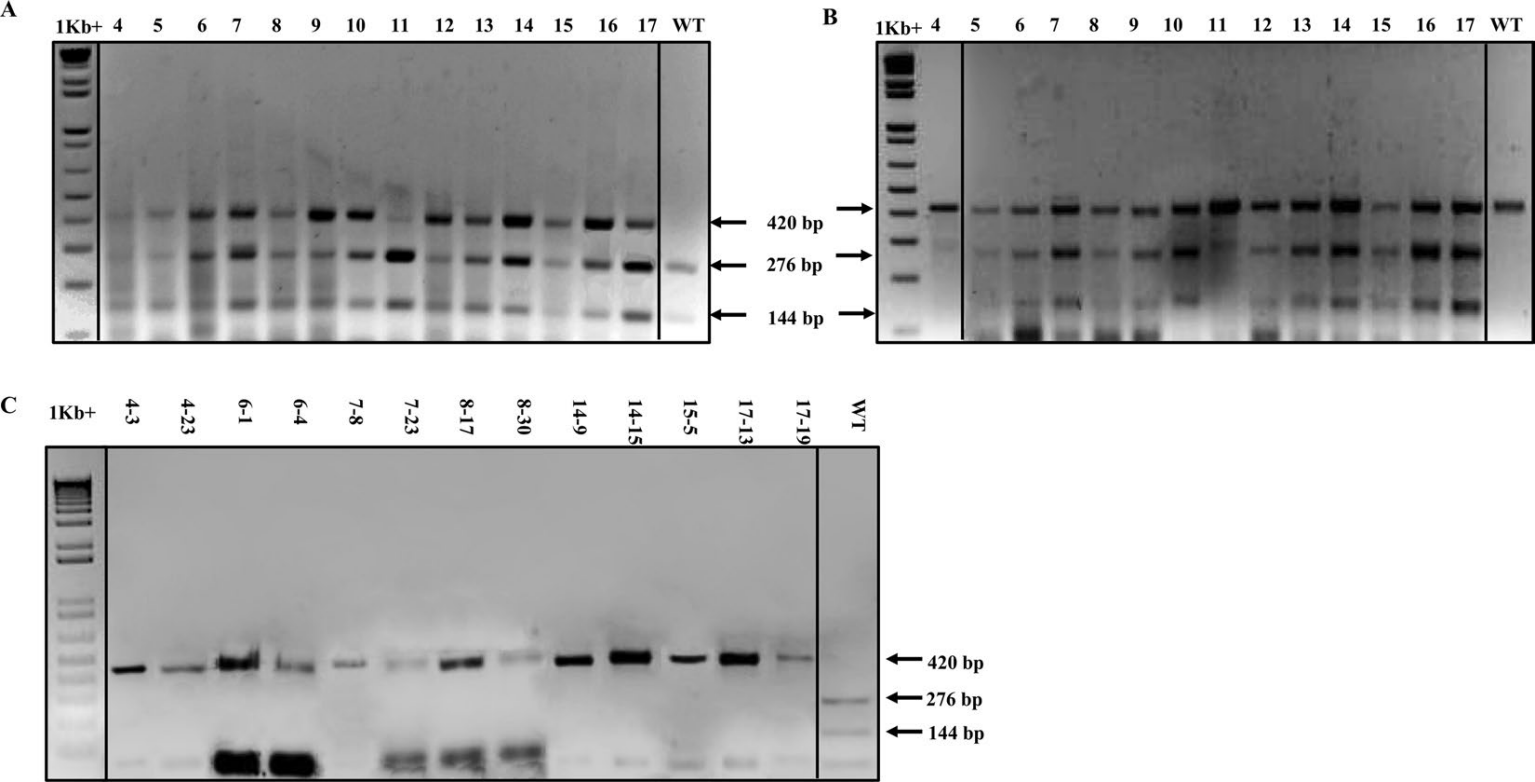
Screening of *At2OGO* mutants created by pRD207 CRISPR gene editing vector. (**A**) RFLP assay by *Bgl*II on WT and T_1_ plants. (**B**) T7E1 assay on WT and T_1_ plants. (**C**) RFLP assay by *Bgl*II to screen for homozygous T_2_ mutants. The 420 bp band indicates mutation of the target site while the 144 bp and 276 bp bands are consistent with products expected from the WT locus. Due to the sample size, the images were grouped from different gels, but all samples in the same image were electrophoresed at the same voltage for the same length of time. The 1Kb+ molecular weight marker and the WT sample were from the same gel.

### Molecular characterization of *At2OGO*-KO (*dmr6*) mutants and identification of homozygous progeny

**Figure 2:**
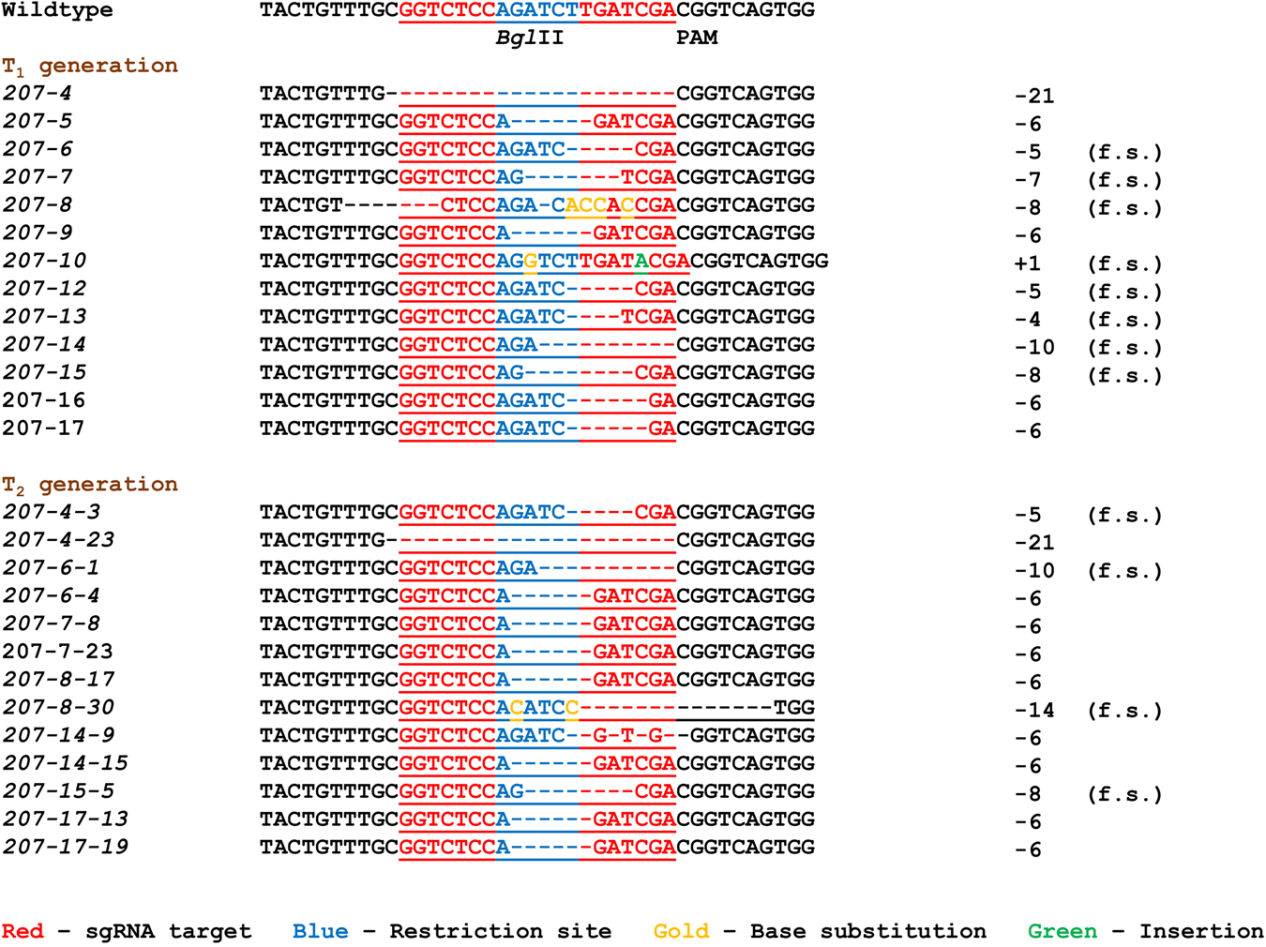
Mutation profiles of *At2OGO*-KO (RD207) mutants at T_1_ and T_2_ generations. “f.s.” indicates frameshifting mutations. “PAM” (CGG) indicates the protospacer adjacent motif for Cas9.

The presence of the Cas9 gene in putative *At2OGO* (pRD207) CRISPR-edited transformants was confirmed by PCR using Cas9 specific primers and genomic DNA extracted from each T_1_ line as the template. Putative CRISPR-edited lines were further characterized by restriction fragment length polymorphism (RFLP) for loss of the *Bgl*II site that resides within the edited target (Figs. 1, 2). Sanger sequencing of PCR products amplified from around the edited region was used to characterize the nature of mutations present in the T_1_ progeny. A 420 base pair (bp) genomic DNA (gDNA) region, flanking the target region, was PCR-amplified and analyzed for the presence or absence of the *Bgl*II restriction digestion (Fig. 1A) and by T7E1 heteroduplex hybridization assay (Fig. 1B). Figure 1A shows that *Bgl*II digestion of PCR products amplified from all of the putative mutant lines gave rise to three DNA fragments. In contrast, *Bgl*II digestion of amplicons from wild type (WT) plants gave rise to only two DNA fragments. The lower two bands (276 bp and 144 bp) represent the digested PCR fragments generated by *Bgl*II digest of unedited alleles while the upper band (420 bp) represents the undigested PCR fragments amplified from edited alleles. The presence of both *Bgl*II-digested and *Bgl*II-undigested products in the lines analyzed indicates that all the RD207 mutants recovered are monoallelic, each containing one edited allele and one WT allele. The complete digestion of the WT amplified PCR product indicates that *Bgl*II was able to completely digest these templates and that the presence of three bands in the mutant lines is not the result of incomplete DNA digestion. That the RD207 plants carry monoallelic mutations was further supported by the results of the T7E1 assay, which revealed three DNA fragments in all mutants, characteristic of both digested and undigested assay products. To confirm the presence and nature of CRISPR/Cas9-edited mutations in the RD207 plants, the undigested PCR amplified fragments straddling the target mutation site were purified and their DNA sequences were determined by Sanger sequencing. T_1_ mutants were selfed and the resulting T_2_ generation was analyzed by RFLP assay to screen for homozygous, biallelic mutants. As shown in Fig. 1C, 13 homozygous T_2_ plants were identified. The presence of a single *Bgl*II-undigested PCR fragment in these T_2_ plants indicate that they all contain biallelic mutations at the *At2OGO* target site.

Figure 2 shows the DNA sequences flanking the gene editing target site for RD207 (*At2OGO*-KO) mutants in both the T_1_ and T_2_ generations. Nine different mutation patterns were observed in the T_1_ mutants. Thirteen out of fourteen T_1_ mutants had nucleotide deletions around the target site while one mutant had an insertion within the target site. 57% (8 out of 14) of the T_1_ mutants were confirmed to have frameshift (f.s.) mutations that would disrupt the active site of *At2OGO*. Most of the mutation patterns from T_1_ generation were retained in the T_2_ homozygous mutants. 31% (4 out of 13) of the identified homozygous T_2_ mutants carried stable frameshifting deletions at the target site. PCR analysis with Cas9-specific primers showed that the 13 frameshifted T_2_ homozygous mutants had lost the gRNA and the Cas9 cassettes through meiotic segregation, indicating that these plants were edited at the target site but otherwise transgene-free.

We chose the homozygous *At2OGO*-KO mutant line RD207-15-5 for subsequent disease assessment and barley gene complementation experiments. The RD207-15-5 mutant carries an 8 bp frameshifting deletion in exon 3 of the *DMR6* gene resulting in the premature termination of the gene product. It is expected that this truncated gene product would lack an intact oxoglutarate/iron-dependent dioxygenase domain at the C-terminal, resulting in the loss of enzymatic activity, congruent with the similar disruption of function in the original *dmr6* point mutant. The reduction of *Hyaloperonospora parasitica* sporangiophores in the *dmr6* mutant^37^ suggested that the RD207-15-5 mutant might also display reduced susceptibility to *Fg* infection.

### Identification of *DMR6* (*2OGO*) orthologue gene from barley cultivar Conlon for complementation test

We searched the PGSB barley genome database for *DMR6* orthologues in barley (*Hordeum vulgare*, *Hv*). Currently, high quality reference genome assembly and automated gene annotation have been carried out only on barley cv. Morex^43^. One copy of the *DMR6* orthologue, *Hv2OGO* gene was identified in cv. Morex. The mRNA and gDNA sequences were annotated as MLOC_59596.1 and HORVU4Hr1G084810.2, respectively, in the database. We focused on *DMR6* orthologues from Conlon because this cultivar is more susceptible to *Fg* than is Morex, and provides a better baseline for determining the effects on susceptibility of inactivating this gene in barley, which is our ultimate goal. In order to characterize the *Hv2OGO* gene sequence in cv. Conlon, as compared to cv. Morex, total RNA from cv. Conlon was extracted and sequenced. The RNAseq data were assembled and aligned with the cv. Morex *Hv2OGO* gene sequence to identify the corresponding Conlon *2OGO* gene sequence. The Conlon *Hv2OGO* nucleotide sequence was shown to be 99.9% identical to the Morex *Hv2OGO* nucleotide sequence except for a single substitution at position 396 of the cDNA sequence, changing from cytosine (C) to thymine (T) base. This nucleotide change changes the corresponding amino acid from phenylalanine to leucine, which is a conservative change and maintains side-chain hydrophobicity. The Conlon *Hv2OGO* gene was cloned into the binary vector pEL103 (kindly provided by Dr. E. Lam^44^,), resulting in pRD331, which was then transformed into EHA105 strain of *Agrobacterium tumefaciens*. The homozygous *At2OGO*-KO (RD207-15-5) plants were transformed with pRD331 using the *Agrobacterium*-mediated floral dip method. Collected T_0_ seeds were screened for germination on 70 μg/L kanamycin-containing medium. A total of 7 putative T_1_ *Hv2OGO*-complement-transformants were recovered from antibiotic selection and integration of the *Hv2OGO* transgene confirmed by PCR amplification of gDNA using *Hv2OGO* gene-specific primers (data not shown). Homozygous *At2OGO*-KO*/Hv2OGO* plants were obtained in the resulting T_2_ generation.

### Characterization of disease phenotype on *At2OGO*-KO and *At2OGO*-KO*/Hv2OGO* homozygous plants

**Figure 3:**
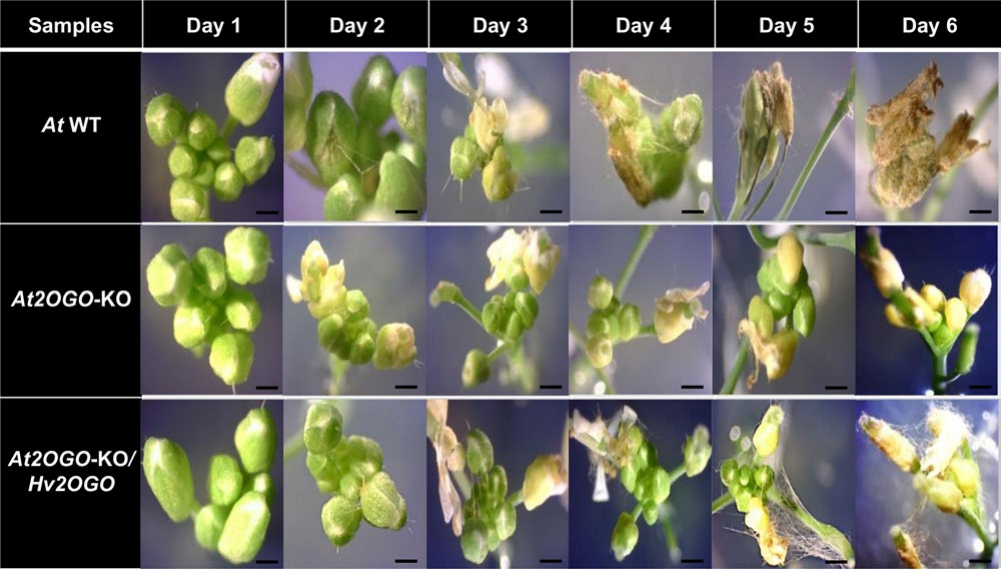
FHB disease progression on detached Arabidopsis inflorescences of *At* WT, *At2OGO*-KO and *At2OGO*-KO/*Hv2OGO* plants. Pictures were taken using the Dino-eye eyepiece camera using a stereomicroscope at 20× magnification. Scale bar = 0.1 mm.

The homozygous *At2OGO*-KO mutant (line RD207-15-5), which contains an 8 bp frameshifting deletion in exon 3 of the *DMR6* gene, as well as homozygous *At2OGO*-KO*/Hv2OGO*-complemented plants (line RD207-15-5/331-1) were used in *Fg* infection assays together with WT Arabidopsis plants as a control. *Fg* infection assays were performed by point inoculation of the sGFP-tagged *Fg* macroconidia onto similarly staged inflorescences detached from 5-week old WT, *At2OGO*-KO and *At2OGO*-KO*/Hv2OGO* Arabidopsis plants followed by stereomicroscopic observation performed each day post inoculation (dpi) for 6 days (Fig. 3). At 2 dpi, fungal hyphae started to grow on the inflorescence surface of both WT and *At2OGO*-KO*/Hv2OGO* plants. Strikingly, *At2OGO*-KO inflorescences remained free from external hyphae growth at this stage. Hyphal growth on WT and *At2OGO*-KO*/Hv2OGO* inflorescences spread from the inoculated inflorescence to neighboring inflorescences starting from 3 to 4 dpi whereas *At2OGO*-KO inflorescences had minimal external hyphal growth on inflorescences at 4 dpi, even at the site of inoculation. At 5 to 6 dpi, the *Fg* hyphae on WT and *At2OGO*-KO*/Hv2OGO* inflorescences became more abundant and started to colonize the entire inflorescence tissue. At this stage the fungus formed a thick mycelial mat and host tissues started to show wilting symptoms. *At2OGO*-KO inflorescences remained in their initial infection condition at 6 dpi, with a minimal amount of external hyphal growth and no observable symptoms in host plant tissues. It is evident from these observations, that the inactivation of the *At2OGO* gene slowed down the infection progress of *Fg*, in concert with its previously established role in conditioning susceptibility to *H. arabidopsidis*. Our results showed that the *At2OGO*-KO inflorescences were more resistant to *Fg* infection than were WT inflorescences. Moreover, the introduction of *Hv2OGO* into RD207-15-5 (*At2OGO*-KO) plants fully restored susceptibility of the host to *Fg*. These results represent a functional complementation of the *At2OGO*-KO by *Hv2OGO* and strongly suggest that *Hv2OGO* may also function as an *Fg* susceptible factor in barley.

**Figure 4:**
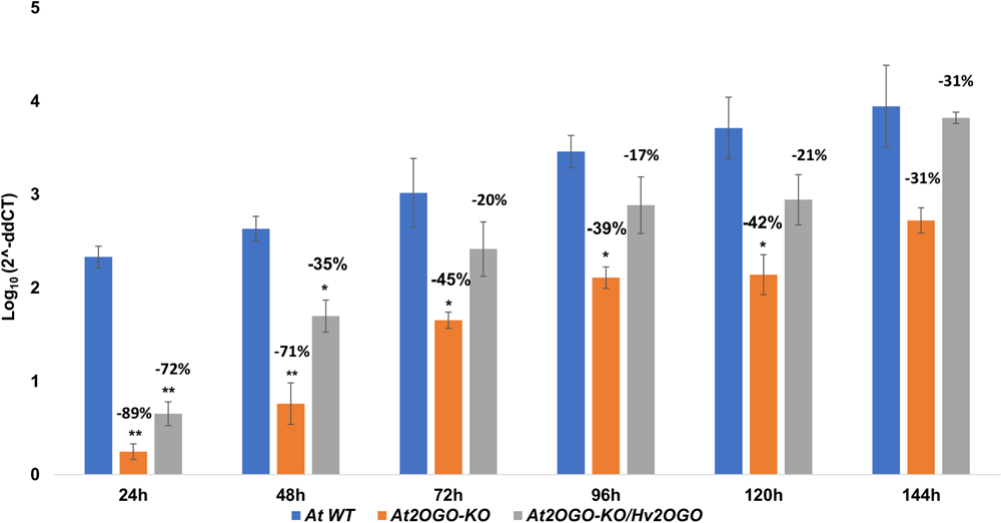
qPCR quantitative analysis of *Fg* growth on inoculated inflorescences. Total DNA was prepared from *Fg*-infected, detached Arabidopsis inflorescences and the amount of *Fg* determined by qPCR using primers specific for the sGFP-transgene present in the *Fg* genome. Experiments were repeated on three independent sample batches. The percentage reduction relative to WT detached Arabidopsis inflorescences is shown within each group of samples collected at a particular time point post inoculation. Statistical significance was determined by *t*-test analysis followed by Holm-Sidak method, with alpha = 0.05. Each time point was analyzed individually, without assuming a consistent SD (p-value: *≤ 0.05; **≤ 0.01; ***≤ 0.001).

**Figure 5:**
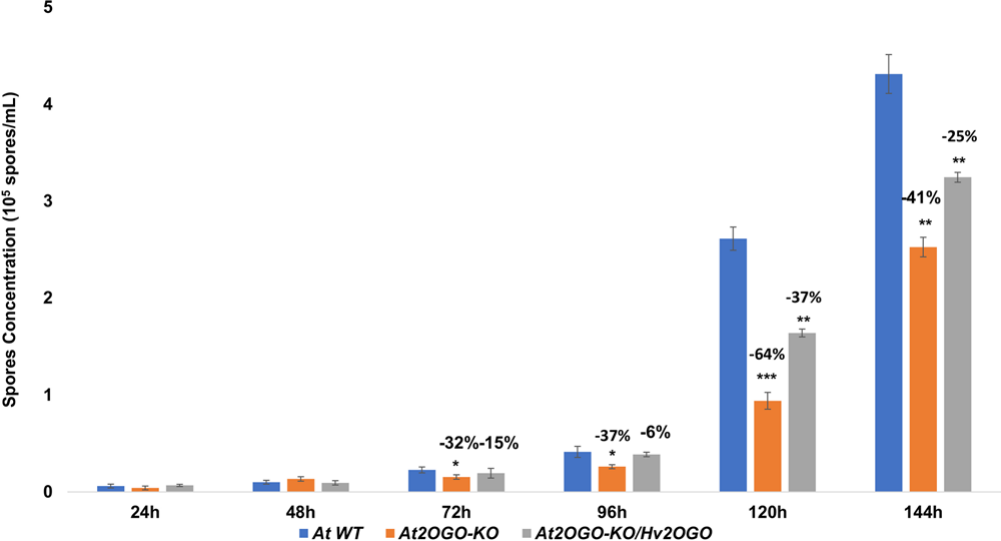
Quantitative measurement of spore production on *Fg* inoculated inflorescences. WT, *At2OGO*-KO and *At2OGO*-KO/*Hv2OGO* lines were inoculated with *Fg* at 0 h and spores collected and determined at the indicated times. The percentage reduction of spore production (compared to WT) is indicated for each of the 72 h, 96 h, 120 h and 144 h time points. The experiment was repeated with three independent batches of samples. Statistical significance was determined by *t*-test analysis followed by Holm-Sidak method, with alpha = 0.05. Each time point was analyzed individually, without assuming a consistent SD (p-value: *≤ 0.05; **≤ 0.01; ***≤ 0.001).

In order to assess the ability of *Fg* to colonize the host plant, we quantitated the amount of *Fg* that had penetrated into the host tissue by qPCR analysis of fungal DNA (Fig. 4). Inoculated inflorescences were collected daily and *Fg* hyphae and macroconidia were washed off plant tissue surfaces using three washes of sterile deionized water accompanied by mild shaking. The rinsed and pat-dried inflorescences were then processed for analysis by qPCR using primers specific for the sGFP gene present in the *Fg* strain^45^ used in these studies. WT and *At2OGO*-KO*/Hv2OGO* inflorescences showed a similar time course of infection with the amount of *Fg* present within inflorescence tissues increasing steadily and consistently, starting at 48 hpi and continuing to 144 hpi. In contrast, colonization of *At2OGO*-KO by *Fg* proceeded at a significantly reduced rate, equivalent to 30–60% that observed in WT or *At2OGO*-KO*/Hv2OGO* complemented plants. Indeed, *Fg* levels in *At2OGO*-KO inflorescences never reached the levels of infection observed in WT or complemented inflorescences within the time course of the experiment. Spore production on *Fg* inoculated inflorescences was quantitated using a hemocytometer (Fig. 5) and provided another readout of the degree of colonization of the plants. These results resembled the qPCR assay in that the spore production increased rapidly in WT and *At2OGO*-KO*/Hv2OGO* inflorescences starting from 96 hpi. Again, in contrast, spore production in *At2OGO*-KO inflorescences was markedly reduced, compared to WT levels, indicating that the KO-mutation of *At2OGO* suppressed *Fg* proliferation and that this deficiency could be complemented by introduction of an orthologous gene from barley. At 144 hpi, spore production in KO and complemented plants were, respectively, 41% and 25% lower than in WT plants. It is worth nothing that the ability of the barley orthologue to complement *At2OGO*-KO is only partial, in that WT levels of *Fg* DNA were not reached until late in infection, at 144 hpi, while the number of spores produced in the complemented line never fully reached those seen in WT plants. Nonetheless, the barley orthologue was clearly able to substantially restore *Fg* colonization of the host, compared to *At2OGO*-KO plants. Together, these results show that *At2OGO* mutants are effective in restricting *Fg* growth and that the barley *Hv2OGO* gene can function to restore susceptibility when expressed in this heterologous plant system.

### Gene expression profiling on plant defense signaling pathway-related genes

**Figure 6:**
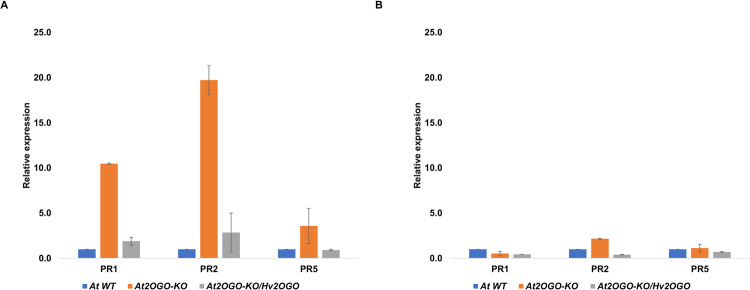
Relative expression of plant defense-related genes by RT-qPCR on non-inoculated leaf (**A**) and floral (**B**) samples of WT, *At2OGO*-KO and *At2OGO*-KO/*Hv2OGO* plants. The experiment was repeated three times with samples from three individual plants. The relative expression values were averaged and standard errors calculated.

The involvement of salicylic acid (SA), jasmonic acid (JA) and ethylene (ET) in the response of WT, *At2OGO*-KO and *At2OGO*-KO*/Hv2OGO* plants to *Fg* was explored by analyzing changes in the expression of genes specific to these signaling pathways. Levels of gene expression were determined by RT-qPCR. *At2OGO* T-DNA-KO plants have been shown to accumulate SA, due to the inability of these mutants to metabolize SA to 2,5-DHBA^36^. Higher levels of SA can induce defense-related genes and this may lead to the heightened resistance observed in our studies and in previous studies of *dmr6* plants inoculated with *Fg*. Previous studies showed that the expression of PR genes, such as *PR1*, in *dmr6* plants was constitutively elevated ~10-fold in healthy, uninfected leaf tissues^38^. Consequently, we analyzed uninoculated floral and leaf samples from the *At2OGO* mutant plants generated here by gene editing. Figure 6A shows that in this study, basal levels of gene expression of *PR1*, *PR2* and *PR5* in *At2OGO*-KO leaf tissues were markedly elevated compared to WT plants. Indeed, we observed an even stronger induction for the *PR2* gene, which was induced ~20-fold over WT levels, compared to the ~10-fold induction observed for this gene in *dmr6* leaf tissues^38^. Surprisingly, uninoculated floral samples of *At2OGO*-KO did not show a similar elevation of basal expression of genes encoding PR-proteins, compared to WT levels (Fig. 6B).

**Figure 7:**
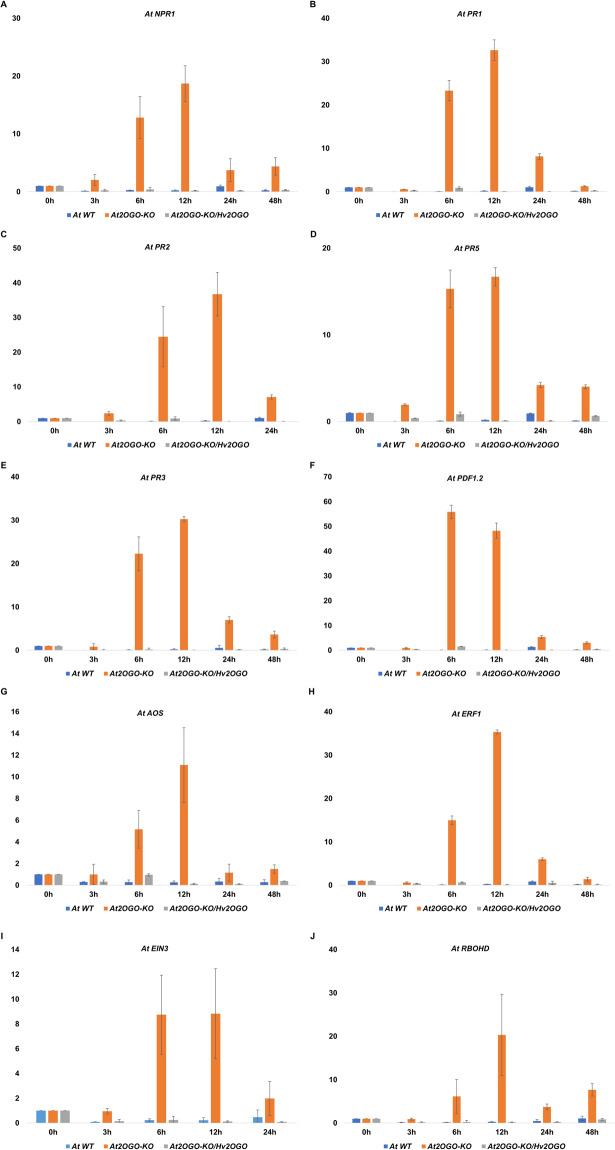
Relative expression of plant defense-related genes by RT-qPCR on *Fg*-inoculated floral tissues of WT, *At2OGO*-KO and *At2OGO*-KO/*Hv2OGO* plants. Relative expression profile includes genes involved in SA signaling (**A**–**D**), JA/ET signaling (**E**–**I**) and ROS activation (**J**). RT-qPCR assay was repeated three times with different batch of samples. The relative gene expression was calculated using the 2^−ΔΔCt^ formula.

Instead, we observed a marked difference in the levels of these genes in floral tissues only when plants were inoculated with *Fg*. For these studies, inflorescences were point-inoculated with sGFP-tagged *Fg* and tissues collected at 3, 6, 12, 24, 48 hpi and processed for RT-qPCR analysis. In contrast to the elevated levels of defense-related genes observed in leaf tissues of *At2OGO*-KO plants, constitutive levels in floral tissue remained near to those seen for WT and complemented plants (Figs. 6 and 7 at 0 h). Surprisingly, *Fg* did not induce defense genes in the inoculated inflorescences from either WT or complemented plants.

In contrast, these genes were massively induced in *Fg-*inoculated *At2OGO*-KO floral tissues. All defense-related genes analyzed were significantly induced by 6 hpi in *At2OGO*-KO inflorescences, compared to either WT or to *At2OGO*-KO*/Hv2OGO* inflorescences (Fig. 7). Figure 7A–D shows relative expression of SA-related genes (*NPR1*) and pathogenesis-related genes (*PR1*, *PR2* and *PR5*) which are key components of SA signaling in plant defense responses to attempted infection. The SA-related gene expression started to increase at 3 hpi and peaked at 12 hpi. *PR3* and *PDF1.2* (Fig. 7E,F), JA and ET responsive genes, were expressed at an extremely high level starting at 6 hpi and decreased after 12 hpi. The *AOS* gene product (Fig. 7G) is involved in the upstream JA signaling pathway. Expression of this gene started to increase at 6 hpi, peaked at 12 hpi and dropped to basal levels at 24–48 hpi. The *EIN3* and *ERF1* genes (Fig. 7H,I), which are activated by ET, displayed a similar gene expression pattern to *AOS*, with induction observed at 6–12 hpi and the expression levels decreasing at 24–48 hpi. These genes were upregulated at 6–12 hpi and decreased to a lower level of expression at 24–48 hpi, except that *PR1* gene expression dropped to the basal level at 48 hpi. The *RBOHD* gene (Fig. 7J) was highly expressed at 6–12 hpi and maintained a much lower expression at 24–48 hpi. Overall, *At2OGO*-KO plants displayed an amplified inducible defense gene response in the inflorescences, which may explain their enhanced disease resistant phenotype when inoculated with *Fg*.

## Discussion

One of the major challenges for agribusiness and food security is to maintain sustainable agricultural practices while increasing both the quantity and quality of food produced. Pathogen attack is the leading cause of both yield loss and decreased crop quality globally. The discovery of disease susceptibility genes has made them an attractive target for gene manipulation since loss-of-function mutations may enhance plant internal resistance to infection. This potentially avoids problems of public acceptance associated with the creation of crops containing transgenes that enhance disease resistance. Moreover, loss-of-function mutations may also provide more resilient resistance under field conditions, since it is more difficult for the pathogen to revert to a virulent form, as is the case when specific R-genes are introduced or when single defense responses are enhanced. For these reasons, we are exploring the use of gene-edited, loss-of-function mutants in disease susceptibility genes as a path for creating stable and acceptable crops with a superior disease resistance profile.

The impairment of Arabidopsis *DMR6* (*2OGO*) gene has been shown to confer broad-spectrum disease resistance^37,38^. This mutant was generated by a common mutagen, ethyl methane sulfonate (EMS), which may have caused mutations in other genes^46^. Therefore, in this study, we took advantage of the targeted mutagenesis afforded by CRISPR/Cas9 gene editing technology to generate *At2OGO*-KO mutants whose disease susceptibility phenotype could be determined upon *Fg* infection of inflorescences. Our *At2OGO-*KO plants were phenotypically similar to WT plants and produced viable seeds. It has been shown that the silencing of orthologous *StDMR6* gene in potato resulted in normal looking plants that were resistant to late blight resistant plants^47^. Our results have shown that *At2OGO*-KO mutants are more resistant to *Fg* infection and proliferation than WT plants. We have also shown that the *At2OGO* orthologue of barley cv. Conlon (*Hv2OGO*) can restore the disease susceptible phenotype in *At2OGO*-KO mutants to almost WT levels, thus verifying its ability to function as a disease susceptibility gene and paving the way for editing *Hv2OGO* gene to study its effect on FHB resistance in barley.

*DMR6* belongs to 2-oxoglutarate Fe(II)-dependent oxygenase (2OGO) superfamily whose members are largely involved in the biosynthesis of plant flavonoids and secondary metabolites^48^. The *DMR6* orthologue in wheat was described as a *flavanone-3-hydroxylase* (*F3H*) gene^49^ whose expression has been shown to increase rapidly during incompatible interaction with Hessian fly, indicating its involvement in insect defense in wheat^50^. The discovery that the loss-of-function *dmr6* mutant can confer broad spectrum disease resistance led us to identify potential homologues or orthologues of *DMR6* in barley, with the goal of manipulating FHB susceptibility in that crop. In order to understand the role of *DMR6* in barley, the cv. Conlon orthologue was identified, cloned and transformed into the *At2OGO*-KO mutant in order to verify whether the barley orthologue carries the same function in facilitating *Fg* invasion. Based on our results using quantitative assays for disease progression, *At2OGO*-KO plants were able to slow down fungal invasion on inflorescence tissues and delay the onset of spore proliferation, compared to WT and to *Hv2OGO-*complemented plants. Gene editing carried out in this study introduced frameshift mutations in exon 3 that truncated the C-terminal Fe(II) 2OG dioxygenase domain of the polypeptide and resulted in a protein that is predicted to lack dioxygenase enzyme activity. The disruption in this domain and the concomitant loss of 2OGO enzyme activity is further expected to affect the biosynthesis and catabolism of plant hormones, such as salicylic acid, ethylene, gibberellins and flavonoids^48^. Biochemical analysis of the 2OGO protein from previous reports indicated that it has a specific function in fine-tuning SA homeostasis^40^. Specifically, 2OGO was shown to convert SA to 2,5-dihydroxybenzoic acid (2,5-DHBA) while a related OGO encoded in Arabidopsis by the *DLO1* gene converts SA to 2,3-dihydroxybenzoic acid (2,3-DHBA)^40^. Mutation of either OGO results in elevated SA levels and this is thought to form the basis for the enhanced, broad spectrum resistance found in either single or double mutants of these genes. These findings suggest that 2OGOs function as plant immunity suppressors for pathogen invasion. In this study, qPCR showed that *At2OGO*-KO floral tissues were able to restrict fungal invasion, compared to either WT or *Hv2OGO-*complemented floral tissues. Even though the more resistant *At2OGO*-KO plants might not have completely blocked the entry of *Fg* into host tissue, fungal progression and proliferation were greatly impeded and infection proceeded at a much slower rate. This observation provides solid evidence for an efficient defense mechanism within the resistant plants and points to the value in trying to enhance this response as an effective defense against FHB and, possibly, other disease as well. We further characterized this enhanced resistance by measuring spore production to measure the *Fg* disease progression and to determine the onset of *Fg* colonization and host wilting. Based on our results, *At2OGO*-KO floral tissues were able to delay the fungal progression for at least 48 hours and restrict spore production by approximately 30–60% compared to the WT and the *Hv2OGO-*complemented floral tissues. It seems that WT and the *Hv2OGO-*complemented floral tissues were compromised at the end of 2 dpi when *Fg* started to overwhelm the host defense system and exploit host nutrients for spore proliferation. On the other hand, *At2OGO*-KO floral tissues managed to strengthen their defense system and alleviate the disease symptoms. Our data showed that impairment of the *At2OGO* gene leads to enhanced FHB disease resistance in Arabidopsis.

In order to better understand the underlying molecular mechanism of the resistant plants, we performed RT-qPCR assay to examine the effect of *At2OGO* mutation on plant defense signaling pathways. In concert with the notion that enhanced resistance in *At2OGO*-KO plants results from elevated SA levels, we found significantly higher, constitutive levels of defense response- and defense-related genes in leaf tissues. However, the steady state levels of these genes in non-*Fg* inoculated floral tissues were not significantly induced, compared to WT plants. Instead, we found that the inducible response to infection was markedly enhanced in *At2OGO*-KO floral tissues, compares to WT or *Hv2OGO-*complemented floral tissues. *Fg*-inoculated floral tissues of *At2OGO*-KO mutant plants showed much higher levels of inducible gene expression in all the defense-related genes analyzed, with the most elevated levels at 3 hpi. In contrast, both WT and *Hv2OGO-*complemented floral tissues displayed much lower levels of gene expression upon fungal infection and showed no significant induction upon infection. These results suggest that enhanced resistance expressed in floral tissues is not the result of the prior activation of defense genes, as seen in leaf tissues. Instead, the ability of the floral tissue to rapidly and extensively react to the presence of the pathogen is markedly enhanced over the WT. The nature of such a priming effect remains to be determined, but may resemble those observed in other SA-related effects such as systemic acquired resistance (SAR)^51,52^. In barley and wheat, floral tissues represent the natural site of primary infection with Fg, fungal penetration occurring on the anthers and proceeding through the floral tissues and into neighboring tissues. It would be interesting to determine if this course of infection results from the opportunistic infection of floral tissues that, for whatever reason, either lack effective basal or inducible defenses.

*DMR6* has been shown to encode a salicylate 5-hydrolase^40^ whose impairment results in the loss of hydrolase activity and the accumulation of high levels of SA which, in turn, stimulate systemic acquired resistance (SAR)^53^. SA is the central player in SAR and known to be a critical defense against biotroph infection. The accumulation of SA can enhance SAR responsiveness and therefore pathogen resistance. Based on this, we expected that during the early hemibiotrophic phase of *Fg* infection, defense responses of *At2OGO*-KO mutants would be expected to respond in a two-fold manner: first, by elevating the expression of SA-related genes, such as *NPR1*, and other SAR-associated markers (*PR1*, *PR2*, *PR3*, and *PR5*); second, by triggering an oxidative burst characterized by the production of reactive oxygen species (ROS). Our results support these predictions. Figure 7A–D showed that SA-related genes were expressed more strongly in *At2OGO*-KO plants, compared to WT, with accumulation starting from 3 hpi and peaking at 12 hpi, followed by a decrease thereafter. *At2OGO*-KO plants are predicted to have elevated levels of SA, as shown previously for *dmr6* mutants. This provides a coherent explanation for the enhanced resistance seen in *At2OGO*-KO plants, based on higher levels of SA leading to either elevated levels of defense gene expression, as seen in leaf tissues, or the potentiation of the defense response, as seen in floral tissues which lack elevated basal defenses but which display a markedly enhanced inducible response. Either approach is likely to play a role in restricting fungal invasion and preventing further proliferation.

Following SA-related gene induction, it has been established that the JA/ET signaling pathways are activated to mediate the resistance against necrotrophic pathogen^54^. Our results have shown the sequential pattern in the activation of the defense pathways. The JA/ET signaling pathways-related genes (*AOS*, *EIN3*, *ERF1*, *PR3* and *PDF1.2*) were induced rapidly starting from 6 hpi and their expression decreased after 12 hpi (Fig. 7E–J). The upregulation of JA/ET-related genes and the marked induction of *PDF1.2* (approximately 56-fold compared to the WT and *At2OGO*-KO*/Hv2OGO* plants) indicate that these genes may also play a role in the enhanced resistance displayed by *At2OGO*-KO plants and that this, in turn, may be responsible for slowing down *Fg* infection and colonization. The *RBOHD* gene was highly expressed from 6–12 hpi, implicating it in regulating ROS production as a protective mechanism during attempted pathogen invasion^55–57^. Based on our qPCR analysis, JA signaling was activated in parallel with the SA signaling to attenuate damage caused by fungal infection, which was similar to responses observed in previous studies^58^. Overall, the much higher level of defense gene expression may be the underlying mechanism responsible for enhanced *Fg* resistance of *At2OGO*-KO plants. The overall pattern appears to involve the SA signaling pathway at the early stage of *Fg* infection followed by the activation of JA signaling pathway at later stages of infection. The impairment of *DMR6* gene in *At2OGO*-KO mutant may be responsible for increasing SA levels which directly exerts its effects on defense responses via the SA/JA/ET signaling pathways. These defense pathways can then act, in turn, to augment natural levels of disease resistance during *Fg* infection. Plants complemented with the barley orthologue *Hv2OGO* were almost fully restored to WT levels of *Fg* susceptibility, strongly suggesting that *Hv2OGO* may be similarly involved in conditioning FHB susceptibility in barley plants. If so, CRISPR-editing the *Hv2OGO* gene in barley cultivars would represent a promising strategy for combating FHB in this, and other crop species.

## Methods and materials

### Arabidopsis thaliana plant growth

*Arabidopsis thaliana* Columbia ecotype (Col-0) seeds were sterilized with 30% bleach for 30 min and washed five times with sterile water. Sterilized seeds were stratified at 4 °C for two days and plated on growth medium [Murashige and Skoog (MS) medium containing 1% (w/v) sucrose and 0.3% (w/v) Gelzan]. The plated seeds grew at 22 °C under 16 h/8 h light-dark photoperiod. Germinated seedlings were transferred to Pro-Mix soil in an environmental-controlled chamber with a 16 h/8 h light-dark photoperiod at 22 °C and 40%-60% relative humidity.

### Construction of *At2OGO* CRISPR-editing vector

The *Arabidopsis thaliana* genomic and mRNA sequence of *2-oxoglutarate and Fe(II)-dependent oxygenase* (*At2OGO*) was obtained from TAIR (https://www.arabidopsis.org/) and NCBI (https://www.ncbi.nlm.nih.gov/) databases with accession number of AT5G24530 and NM_122361.4 respectively. The gRNA target sequence for *At2OGO* gene (5′- GGTCTCC*AGATCT*TGATCGACGG-3) was identified according to selection guidelines (www.addgene.org/crispr/guide) and included with 20 nucleotides upstream of a proto-spacer adjacent motif (PAM, CGG) sequence (5′-N_20_-NGG-3′) and a target sequence beginning with the G nucleotide base that includes a *AGATCT Bgl*II restriction site. The *At2OGO-*editing vector was constructed following previously published protocols^29^. The gRNA sense and anti-sense oligonucleotides (forward: 5′- GATTGGTCTCCAGATCTTGATCGA-3′; reverse: 5′- AAACTCGATCAAGATCTGGAGACC-3′) were phosphorylated and annealed under the following conditions: 37 °C for 30 min, 95 °C for 5 min and then ramping down to 25 °C at the rate of 5 °C/min, in a 10 μl reaction (1 μl of 10 × T4 ligation buffer, 0.5 μl of T4 PNK, 1 μl of each oligo at 100 μM). The annealed gRNA oligonucleotides were cloned into psgR-Cas9-At vector (kindly provided by Dr. J. Zhu^28^,). The resulting plasmid was sequenced and confirmed followed by subcloning into the plant expression vector pCAMBIA1300 (https://www.addgene.org/vector-database/5930/), resulting in pRD207, and introduced into *Agrobacterium tumefaciens* EHA105 strain for subsequent plant transformation.

### *Arabidopsis thaliana* plant transformation and mutant selection

*Arabidopsis thaliana* Col-0 plants were grown for 3–4 weeks until flowering. *Agrobacterium tumefaciens* EHA105 strain harboring pRD207 (*At2OGO* gRNA/Cas9) was grown in 2 ml of LB liquid medium containing 50 μg/ml kanamycin and 50 μg/ml chloramphenicol. The 2 ml overnight culture was inoculated into 200 ml LB liquid medium containing the same antibiotics and was grown in a shaking incubator at 30 °C for approximately 5 h until the OD_600_ reached to 0.6. *Agrobacterium* cells were collected by centrifugation and rinsed twice with 5% sucrose before resuspending into 300 ml of 5% sucrose solution containing 0.05% Silwet L-77. Floral buds were dipped into *Agrobacterium* solution for 1–2 s^59^. The inoculated plants were covered with a clear plastic dome for 24 h before returning to growth chamber. The transformed plants were grown for another 3–4 weeks for seed harvesting. The collected T_0_ seeds were sterilized by 30% bleach and selected on MS growth medium containing 50 μg/ml hygromycin. The selected mutant plants were transferred to soil and acclimated growth in the growth chamber.

### Genomic DNA (gDNA) isolation of Arabidopsis mutant plants

200 mg leaf tissues from each putative mutant plant were collected for gDNA isolation using CTAB extraction buffer (2% cetyl trimethylammonium bromide, 1% polyvinyl pyrrolidone, 100 mM Tris-HCl, 1.4 M NaCl, 20 mM EDTA). The CTAB-leaf tissue mix was vortexed vigorously for 30 s and then incubated at 65 °C for 1 h followed by phenol-chloroform isolation. The supernatant was then mixed with 100% ethanol to precipitate the DNA followed by a clean-up step with 70% ethanol. After centrifugation, DNA pellets were air-dried and resuspended in sterile deionized water. The concentration of DNA was measured with a Nanodrop spectrophotometer (Thermo Fisher, Waltham, MA, United States).

### Mutant analysis: mutant confirmation, restriction fragment length polymorphism (RFLP) and T7 endonuclease 1 (T7E1) assays

The presence of the Cas9 gene in putative mutant plants was confirmed by PCR-amplification using Cas9 primers (forward: 5′- GAAGCGGAAGGTCGGTATCCACGG-3′; reverse: 5′- GGCCAGATAGATCAGCCGCAGGTC-3′) for its integration into the genome. The gDNA samples were then used to amplify a 420 bp fragment flanking the target sequence (forward: 5′- GTGCTTGGTGAACAAGGTCAACAC-3′; reverse: 5′- GTGCTTGGTGAACAAGGTCAACAC-3′). The amplified fragments were purified using phenol/chloroform clean-up method and resuspended in deionized water prior to analysis. RFLP assay was carried out by incubating *Bgl*II restriction enzyme with the purified 420 bp PCR fragment from each putative mutant line and the WT plant. Digested samples were electrophoresed on 1% agarose gel and the gel images were captured under UV illumination. Putative mutants were also analyzed using the T7E1 assay, in which formation of a heteroduplex between WT and mutant PCR products is followed by digestion of mismatched nucleotides by T7E1^60^. The purified 420 bp PCR fragments were denatured and annealed in a thermocycler using the following program: 95 °C for 5 min, ramping down to 85 °C at the rate of 2 °C/s, 25 °C at the rate of 0.1 °C/s. The mixture was then digested with 0.5 μl of T7E1 enzyme (10 U/μl) at 37 °C for 60 min. Samples were electrophoresed on 1% agarose gel immediately after incubation and images captured under UV illumination.

### Complementation of *At2OGO*-KO line with *Hv2OGO*

In order to clone the *Hv2OGO* cDNA from barley cv. Conlon, total RNA was isolated from four-week-old leaf tissues and subjected to RNAseq analysis by Novogen Co., Ltd.. The RNAseq data has been deposited in NCBI SRA database (accession number SRR10059574) under BioProject PRJNA563590. The RNAseq data from Conlon were aligned to cv. Morex *2OGO* gene sequence (mRNA: MLOC_59596.1; gDNA: HORVU4Hr1G084810.2) using the NCBI BLAST tool (https://blast.ncbi.nlm.nih.gov/Blast.cgi). Conlon *Hv2OGO* cDNA was amplified by PCR with gene specific primers (forward: 5′- GGTCTAGAATGGCGGAGCAGCTCATCTC-3′; reverse: 5′- GGGAGCTCCTAGGTTCTGAAGAGCTCCAGGC-3′) and cloned into plant expression vector pEL103 (kindly provided by Dr. E. Lam^44^,), resulting in pRD331, which was then transformed into *Agrobacterium tumefacien* EHA105 strain. The homozygous *At2OGO*-KO line (RD207-15-5) was complementarily transformed with pRD331 *via* the floral dip method. Seeds from T_0_ plants were selected on 70 μg/ml kanamycin. Segregation of kanamycin resistance in the confirmed T_1_ complemented plants was followed by plating seeds on 70 μg/ml kanamycin. Homozygousity of the T_2_ generation was similarly confirmed by antibiotic screening of segregants.

### *Fg* inoculation on detached Arabidopsis inflorescences and quantitative assays for disease development

*Fg* tagged with sGFP (superfolder green fluorescent protein)^45^ was cultured on potato dextrose agar (PDA) for 7 days at 22 °C under UV light for 24/7. Fungal plug was extracted from PDA plate and cultured in 50 ml of mung bean soup for another 7 days to generate macroconidia^61^. The macroconidia were filtered out from mung bean soup using Miracloth (Millipore Sigma, Burlington, MA, United States) and washed twice with sterile deionize water before resuspension in sterile water. Macroconidia were counted using hemocytometer. Macroconidia were resuspended to an inoculum of 1 × 10^6^ spores/ml. Bolting floral buds were detached from Arabidopsis plants and laid perpendicularly onto 0.7% water agar in petri dishes. Each inflorescence was inoculated with 2 μl of 1 × 10^6^ spores/ml macroconidia solution along with a duplicate set of mock sample that was inoculated with sterile water. The inoculated detached inflorescences were maintained in a 100% humidity condition by spraying water on the inner side of the petri dish lids prior to plate sealing. The plates were left in environmentally controlled chamber with a 16 h/8 h light/dark photoperiod at 22 °C. Images were taken using the Dino-eye eyepiece (Dino-Lite, Hsinchu, Taiwan) attached to the Nikon SMZ 645 stereomicroscope (Nikon corp., Minato, Japan). Samples were collected every 24 h by pooling four inflorescences in a tube per time point until reaching 144 hours. After each collection, the pooled samples were rinsed with 1 ml of sterile water and mild shaking to remove *Fg* spores from the surface of inflorescence. At each time point, the first 1 ml of rinsed water was collected to quantitate the spore production at each time point using a hemocytometer. Pooled samples were rinsed for another two times with 1 ml of water to remove any surface-attached spores. Total DNA from these pooled samples were then isolated using the CTAB method. The amount of *Fg* DNA present in pooled samples was determined by quantifying the levels of the *Fg*-borne sGFP transgene. Quantitation of sGFP was performed by RT-PCR using an Applied Biosystems StepOne Plus thermocycler (Applied Biosystems, Thermo Fisher, Foster City, CA, United States) and primers specific for the *Fg* sGFP gene (forward: 5′- GTCCGCCCTGAGCAAAGA-3′; reverse: 5′- TCCAGCAGGACCATGTGATC-3′). Both quantitative assays were performed in triplicate and averages and standard deviations (SD) determined. Data collected from WT vs. *At2OGO*-KO and WT vs. *At2OGO*-KO*/Hv2OGO* groups were analyzed by Student’s *t*-test analysis using GraphPad software. Statistical significance was determined using the Holm-Sidak method, with alpha = 0.05. Each time point was analyzed individually, without assuming a consistent SD.

### Relative gene expression profiling on plant defense mechanism

In order to understand the molecular mechanisms underlying the defense signaling in *At2OGO*-KO and *At2OGO*-KO*/Hv2OGO* plants infected with *Fg*, some of the key gene targets in the SA, JA, ET and ROS signaling pathways were selected for quantitation by RT-qPCR analysis. Arabidopsis *actin-2* (*ACT2*) gene was used as the reference housekeeping gene in these studies. Primers for each selected gene were designed using the Primer Express software (Applied Biosystems, Thermo Fisher, Foster City, CA, United States). The primer sets for each gene were first validated to have similar amplification efficiency as the *ACT2* gene. Detached inflorescences were each inoculated with 2 μl of *Fg* macroconidia solution as described above, and collected at 3, 6, 12, 24, 48 h time point. Four inflorescences were pooled into a tube and rinsed three times with 1 ml of sterile water. Total RNA was isolated from pooled samples were isolated using the TRIZOL method (Ambion Life Technologies, Thermo Fisher, Carlsbad, CA, United States) and the concentration was measured using the Nanodrop spectrophotometer (Thermo Fisher, Waltham, MA, United States). Reverse transcription (RT) reaction was carried out with the High Fidelity cDNA Synthesis Kit (Applied Biosystems, Thermo Fisher, Foster City, CA, United States) using approximately 2 μg RNA. cDNA generated in these RT reactions were used as templates in RT-qPCR reactions with the SYBR Green 2X Master Mix (Applied Biosystems, Thermo Fisher, Foster City, CA, United States). The RT-qPCR assay was run on the default setting at 95 °C for 3 min for initial denaturation and 40 cycles at 95 °C for 30 s followed by 60 °C for 30 s. The fold-change of gene expression was calculated by the 2^−ΔΔCt^ method^62^. The RT-qPCR analysis was repeated three times with different batches of samples, and the gene expression levels were averaged. Primers used in the RT-qPCR gene expression assay are listed in Supplementary Table 1.

## Supplementary information

Supplementary Information.
