# Supplementary material for: Validation of barley 2OGO gene as a functional orthologue of Arabidopsis DMR6 gene in Fusarium head blight susceptibility
Source: Sci Rep. 2020 Jun 18;10:9935. doi: 10.1038/s41598-020-67006-5 (PMC7303206; doi:10.1038/s41598-020-67006-5)
Supplement: Supplementary file 1 — Supplementary Information. [file 41598_2020_67006_MOESM1_ESM.pdf]

# **Validation of barley *2OGO* gene as a functional orthologue of Arabidopsis *DMR6* gene in *Fusarium* head blight susceptibility**

Yee Chen Low, Michael A. Lawton, Rong Di

Department of Plant Biology, Rutgers University, New Brunswick, NJ, USA

Correspondence should be addressed to R.D. (email: rongdi@sebs.rutgers.edu)

**Supplementary data**

| RT-qPCR primers | Forward | Reverse |
|---|---|---|
| ***NPR1*** | GCCGCCGAACAAGTACTCA | GCTGTTGGAGAGCAATTGCA |
| ***PR1*** | GTCTCCGCCGTGAACATGT | CGTGTTCGCAGCGTAGTTGT |
| ***PR2*** | GCTGGACAAATCGGAGTATGC | CCGATGGACTTGGCAAGGTA |
| ***PR5*** | AACGGCGGCGGAGTTC | CGCCATCGCCTACTAGAGTGA |
| ***PDF1.2*** | TTTGCTTCCATCATCACCCTTA | GCGTCGAAAGCAGCAAAGA |
| ***AOS*** | CCACCGGTTACGGCTCAA | GCGTCGTGGCTTTCGATAA |
| ***ERF1*** | CCCTTCAACGAGAACGACTCA | TTGCGTGGACTGCTCGATT |
| ***EIN3*** | CCGACTCCTCATACCTTGCAA | CGCAGACAAAAGCGATCCA |
| ***PR3*** | ACGCAGTGATCGCTTTCAAA | TGGGAGGCTGAGCAGTCATC |
| ***RBOHD*** | CATGCGGGTGCCCATT | ATCCGCGGCAATTAAACG |
| ***Actin2*** | GATTCAGATGCCCAGAAGTCTT | TGGATTCCAGCAGCTTCCAT |

**Supplementary Table 1.** List of primers used in the RT-qPCR gene expression assay.
